# Effect of vitamin D supplementation on motor symptoms in Parkinson’s disease: a meta-analysis of randomized controlled trials

**DOI:** 10.3389/fnut.2025.1500875

**Published:** 2025-06-09

**Authors:** Jing Xu, Jia Li, Ya-juan Sun, Wei Quan, Yi-dan Qin, Jia Song, Jia-jun Chen

**Affiliations:** Department of Neurology, China–Japan Union Hospital of Jilin University, Changchun, Jilin, China

**Keywords:** Parkinson’s disease, vitamin D, motor symptoms, meta-analysis, randomized controlled trial

## Abstract

**Introduction:**

Lower serum vitamin D levels may associate with higher motor symptom severity in Parkinson’s disease (PD). This study aimed to test the efficacy of supplemental vitamin D on ameliorating motor symptoms in PD, which is the most comprehensive study to assess the relationship between vitamin D supplementation and PD motor symptoms to date.

**Methods:**

An electronic literature search supplemented by hand searching up to Sep 2024 identified 8 randomized controlled trials involving 646 cases of PD. Weighted mean difference (WMD) and 95% confidence interval (CI) of PD were assessed through pooling the collected data from eligible studies using Stata software.

**Results:**

The results indicated that supplemental vitamin D did not reduce the Unified Parkinson’s Disease Rating Scale part III score (WMD=-0.56, 95% CI=[-2.34, 1.23]), 10/8 m walk test time (WMD=0.59, 95% CI=[-0.46, 1.64]) and timed up and go (TUG) test time (WMD=-0.57, 95% CI=[-1.45, 0.31]). A statistically significant benefit of supplemental vitamin D was observed on 6-Minute walking test distance (WMD=24.85, 95% CI=[6.54, 43.16]).

**Discussion:**

This meta-analysis suggested that supplemental vitamin D may extend 6-Minute walking test distance, improve partial motor symptoms. Vitamin D supplementation may play an active inhibitory role in the mechanisms of the development of PD

## 1 Introduction

Parkinson’s disease (PD) is a multisystem clinical syndrome with a range of causes and clinical presentations (1). Motor dysfunction severely affects the quality of life of patients, however, the exact mechanisms underlying neurodegeneration in PD are not fully elucidated, there is no available therapy to slow down or arrest the progression of PD. But many studies have indicated that vitamin D metabolism may be directly or indirectly related to the pathogenesis of PD (2). Vitamin D supplementation may be a promising strategy which is currently being tested for its disease-modifying potential (3–7), especially in younger patients (4). Higher frequency of vitamin D deficiency was observed in PD patients, compared to controls (8). What is more, genetic polymorphisms might induce vitamin D deficiency despite enough sun exposure and rich in vitamin D food intake, enhancing inflammation, there by influencing PD pathophysiology (9). Vitamin D is a hormone rather than a nutritional vitamin that exerts a regulatory role in the pathophysiology of Parkinson’s disease (9, 10). In neurons, vitamin D plays key roles in the suppression of oxidative stress, inhibition of inflammation, neuroprotection, downregulation of inflammatory mediators, and upregulation of many neurotrophins (11). The vitamin D deficiency may involve in the loss of dopaminergic neurons in Parkinson’s disease (10). An analysis of 69,010 patients with incident PD suggested that reasonable ultraviolet B exposure is associated with a lower PD risk in younger persons, and lower serum vitamin D levels are consistently associated with higher motor symptom severity in PD; simultaneously, reduced mobility in advanced disease may result in limited sun exposure and lower vitamin D levels aggravated the disease (5).

The natural sources of vitamin D include sunlight exposure, diet, and vitamin D supplements. Approximately 20% of vitamin D is obtained from food, while the remainder is obtained from ultraviolet radiation inducing skin synthesis of Vitamin D as a product of skin 7-dehydrocholesterol trans-formations (12). The enzyme 1-alpha-hydroxylase metabolizes 25 (OH) D to its active form: 1α,25-dihydroxyvitamin D3 (1,25 (OH)2D3; also known as calcitriol), and the biological functions of 1,25 (OH)2D3 are mediated by the vitamin D receptor (VDR) (13). VDR and metabolic enzymes are abundantly expressed in the substantia nigra (14). Prior studies have indicated that Vitamin D3 may help promote the recovery of dopaminergic function in injured nigrostriatal neurons in rats (3), modulating autophagy (15), and protect dopaminergic neurons against neuroinflammation oxidative stress and in hemiparkinsonian rats (13, 16). Various cell culture studies have shown that calcipotriol can prevent *α*-synuclein aggregate formation and ameliorate PD pathogenesis by raising the intraneuronal free Ca (II) in the brain by promoting the expression of calbindin-D28k at the transcriptional level (6). Vitamin D3 may enhance the expression of key neurotrophic factors, antioxidant markers (17) and interact with tyrosine hydroxylase, Nrf2 to play its neuroprotective actions (18). Vitamin D stimulates the expression of VDR, a transcription factor that is believed to be responsible for the upregulation of microtubule-associated protein 2 and neurofilament heavy polypeptide genes (19). VDR and other nuclear hormone receptors also play a crucial role in myelination, promoting oligodendrocyte maturation and development and preventing demyelination processes (20).

In 2017, The National Institute for Health and Care Excellence guidelines in England advised individuals with PD to take vitamin D supplements (21). Supplemental vitamin D at a dose of 700–1,000 IU per day reduces the risk of falling among older individuals (22). Vitamin D3 significantly prevents the deterioration of the HY stage (23), improving the neuromuscular or neuroprotective function (24), while promoting balance in younger patients with PD (4). However, some studies have suggested that a lowered 25-hydroxyvitamin D concentration is not associated with the risk of PD (25, 26), and chronic vitamin D insufficiency does not threaten dopaminergic system integrity or contribute to PD pathogenesis (27), vitamin D supplementation did not improve the patient’s motor function (28, 29). This meta-analysis aimed to assess the efficacy of vitamin D supplementation in ameliorating motor symptoms in PD.

## 2 Materials and methods

### 2.1 Literature search and inclusion criteria

This study included only randomized controlled trials (RCTs) investigating the effects of vitamin D supplementation or sunlight exposure on motor symptoms in patients with PD. Motor symptom severity was measured using the Unified Parkinson’s Disease Rating Scale Motor Examination Part III (Motor Examination) (UPDRS III), the timed up and go test (TUG), 10/8 m walk test time and 6-Minute walking test distance. The TUG measures the time taken (in seconds) for a patient to stand up from an armchair (with the upper extremities not on the assistive device, but only nearby), walk a distance of 3 m, turn, walk back to the chair, and sit down. The time is started when the test administrator says “go” and is stopped when the patient’s buttocks touch the seat (30). Only RCTs clearly reporting the patient inclusion and exclusion criteria; the process of randomization; the method, dosage, and duration of vitamin D supplements or sunlight exposure; and a comprehensive assessment of UPDRS III, TUG, 10/8 m walk test time and 6-Minute walking test distance were considered.

We searched for RCTs published from January 1, 1983 to Sep 1, 2024, in the PubMed, Embase, Web of Science, Chinese National Knowledge Infrastructure, and Wanfang Medicine electronic databases following the Preferred Reporting Items for Systematic Review and Meta-Analysis guidelines. We used the following Medical Subject Headings (MeSH) keywords as search terms: “vitamin D [MeSH],” “cholecalciferol,” “ergocalciferol,” “25-hydroxyvitamin D,” “25 (OH)D,” “vitamin D analog,” “dihydrotachysterol,” “hydroxycholecalciferol,” “calcifediol,” “calcidiol,” “1,25-dihydroxyvitamin d,” “1-*α*-hydroxyvitamin d,” “1-alpha-hydroxyvitamin d,” “calcitriol,” “alfacalcidol,” “paricalcitol” and “Parkinson’s disease [MeSH],” “Parkinsonism,” “Parkinsonian disorder,” “PD,” “paralysis agitans,” “Lewy body.” Variations or synonyms of the keywords were also used to ensure that a comprehensive search was performed, including terms related to randomized controlled trial. To identify potentially relevant studies that were missed by the search strategy, the reference lists of the retrieved articles were manually screened. Restrictions to reference human studies and those published in English were imposed during reference selection.

### 2.2 Quality assessment and levels of evidence

Studies were critically evaluated for design and risk of bias according to the criteria set out in the Cochrane Handbook for Systematic Reviews of Interventions (31). Each RCT was assessed according to the following seven criteria: (1) sequence generation; (2) allocation concealment; (3) blinding of participants and personnel; (4) blinding of outcome assessment; (5) incomplete outcome data; (6) selective outcome reporting; and (7) other sources of bias.

### 2.3 Data handling and statistical analysis

The eligible studies selected for the meta-analysis were carefully independently evaluated by two reviewers. Disagreements were resolved through consensus. The data retrieved from the studies included the name of the first author, publication year, country, number of cases and controls, and characteristics of the study participants, including age, sex, treatment methods, study duration, 25 (OH) D serum concentration, UPDRS III, TUG, 10/8 m walk test time and 6-Minute walking test distance.

For continuous outcome data, means and standard deviations were used to calculate the weighted mean difference and 95% confidence intervals (CIs) in the meta-analysis. Heterogeneity was evaluated using the Chi-square-based Q statistic (with a level of significance of *p* < 0.1), and its extent was quantified with the *I^2^* statistic; a fixed-effects pooled analysis method was used to combine the results if no or low heterogeneity existed (*I^2^* < 50%), or a random-effects meta-analysis was performed. Stata version 14.0 statistical software was used to perform the meta-analysis and to create forest plots, while quality assessment was performed using RevMan software version 5.3. The ID is CRD42022301110 in PROSPERO.

## 3 Results

### 3.1 Summary of the included studies

**Figure 1:**
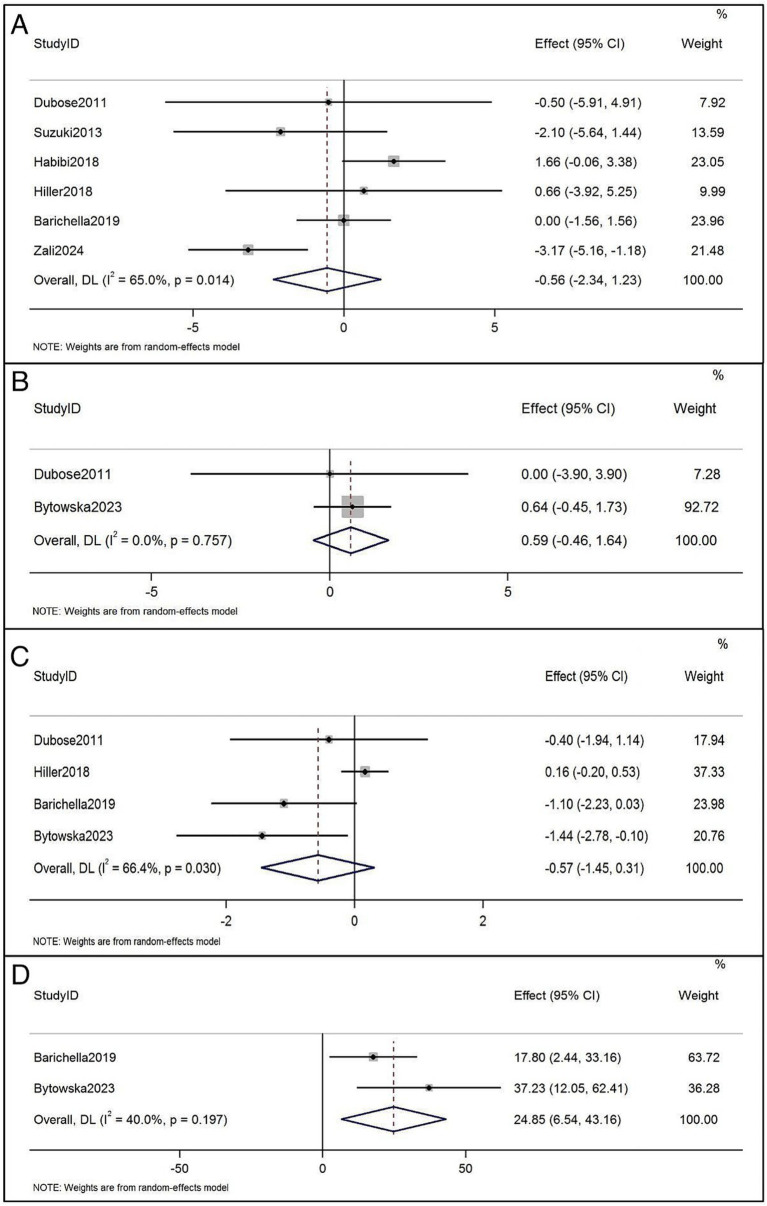
Flow diagram of the literature search protocol, and the identification of relevant studies.

**Figure 2:**
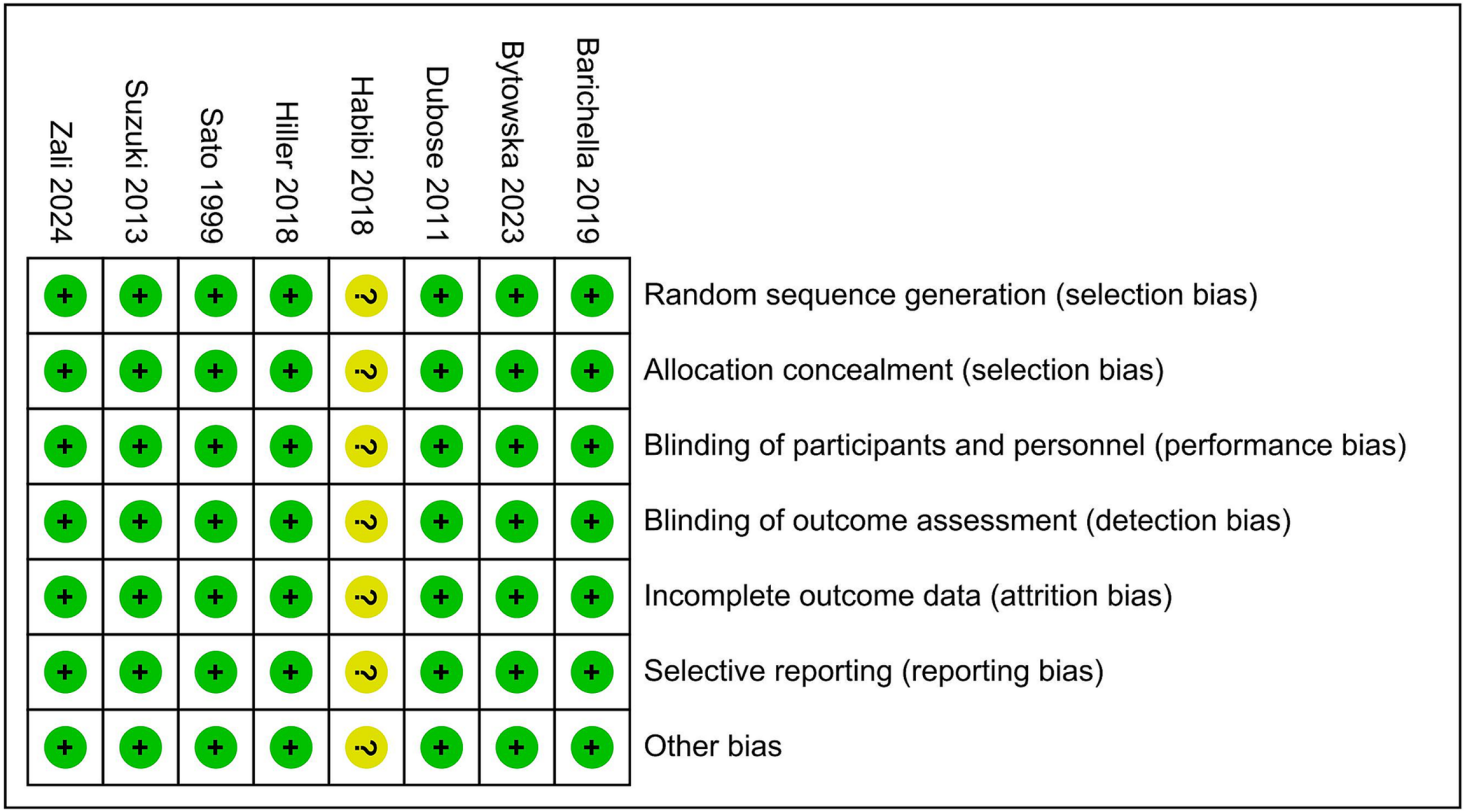
Quality assessment of the included randomized controlled trials using the Cochrane Handbook for Systematic Reviews and Interventions.

A flowchart showing the study selection process is presented in Figure 1. A total of 541 publications were initially identified according to the search criteria. The titles and abstracts of 454 publications were reviewed after removing duplicate studies, and a further 442 non-relevant publications were excluded. For the remaining 12 publications, the full texts were retrieved for further assessment, and 8 RCTs involving 646 cases of PD were included in the qualitative and meta analyses (4, 23, 28, 32–36). Six studies assessed vitamin D with or without calcium, one with probiotics, while one study assessed whey protein–based nutritional formula enriched with leucine and vitamin D. Dubose’s study (33) evaluated patients at 3 and 6 months, and the patients were divided into ON and OFF groups; where the “ON” medication state indicates that the patient shows a good response to medication and minimal symptoms, and the “OFF” medication state describes when medication is not working, we selected the ON groups at 6 months as object of study. The RCTs were published between 1999 and 2024, and had sample sizes ranging from 30 to 150. The mean age of the patients was 63.6 years, and the treatment strategy and other characteristics of the 8 RCTs are presented in Table 1, two of which were conducted in the USA, one in Italy, two in Iran, one in Poland and two in Japan. Except for Sato1999 (35), Habibi2018 (34) (No detailed data) and Zali (36) (no detailed data), 25 (OH) D concentration statistically increased at the end of studies, however, 1, 25-[OH]2D increasing significant in Sato1999 (35). The quality assessment of the included randomized controlled trials is shown in Figure 2. All studies were considered to be of high quality, except one (34).

**Table 1:** Main characteristics of eligible studies for the analysis of vitamin D supplementation in Parkinson’s disease.

Studies, country	Number		Age [year; mean (SD)]		Sex male (%)		Treatment		Adherence	Study duration (m)	25(OH)D serum concentration changes at baseline and endpoints [ng/mL; mean (SD)]	
	T	C	T	C	T	C	T	C			T	C
Sato et al. (35) Japan	43	43	70.5 (2.9)	70.7 (3.3)	18 (41.9)	17 (39.5)	1 μg 1α (OH)D3/d	Placebo	93%	18	11.0 (5.9)	11.8 (6.9)
											11.3 (4.3)	11.6 (6.4)
Dubose et al. (33) USA	16	14	64 (7.9)	65 (7.3)	11 (68.8)	8 (57.1)	Vit. D3 50,000 IU/w + Vit. D 600 IU/d	Placebo+Vit. D 600 IU/d	90%	0	20.2 (8.6)	24.9 (8.6)
										3	69.1 (41.7)	25.9 (9.1)
										6	71.9 (38.3)	25.5 (7.5)
Suzuki et al. (23) Japan	56	58	72.(6.6)	71.2 (6.9)	31 (52)	29 (53)	Vit. D3 1,200 IU/d	Placebo	91%	12	22.5 (9.7)	21.1 (8.8)
											41.7 (12.6)	21.4 (9.8)
Habibi et al. (34) Iran	60	60	44.02 (13.2)	49.9 (11.4)	na	na	Vit. D3 1,000 IU/d	Placebo	na	3	na	na
Hiller et al. (4) USA	28	30	64.63 (8.1)	68.75 (7.6)	23 (82.1)	20 (66.7)	Vit. D 10,000 IU/d + 1,000 mg Calcium/d	Placebo+1,000 mg Calcium/d	88%	4	30.33 (5.378)	29.92 (6.324)
											61.1 (na)	27.8 (na)
Barichella et al. (30) Italy	75	75	66.8 (8.2)	68.5 (7.9)	50 (66.6)	46 (61.3)	Vit. D 1,600 IU/d + 1,000 mg Calcium/d + 40 g of whey proteins+hospital diet	Hospital diet	91%	1	6.6 (14.1) changes	−3.2(−13) changes
Bytowska et al.(32) Poland	21	21	63 (9)	66 (6)	6	13	Vit. D3 4,000–6,000 IU/d	Placebo	69%	3	25.55 (8.94)	21.98 (10.91)
											34.99 (12.27)	<21.98(na)
Zali et al. (36) Iran	23	23	56.34 (10.23)	55.73 (10.98)	14 (60.9)	15 (65.2)	Probiotics + 400 IU Vit. D	Placebo	100%	4	na	na

T, treatment group; C, control group; 25 (OH) D, 25-hydroxyvitamin D; na, not available; Vit. D, vitamin D.

### 3.2 Effects on motor symptoms

**Figure 3:**
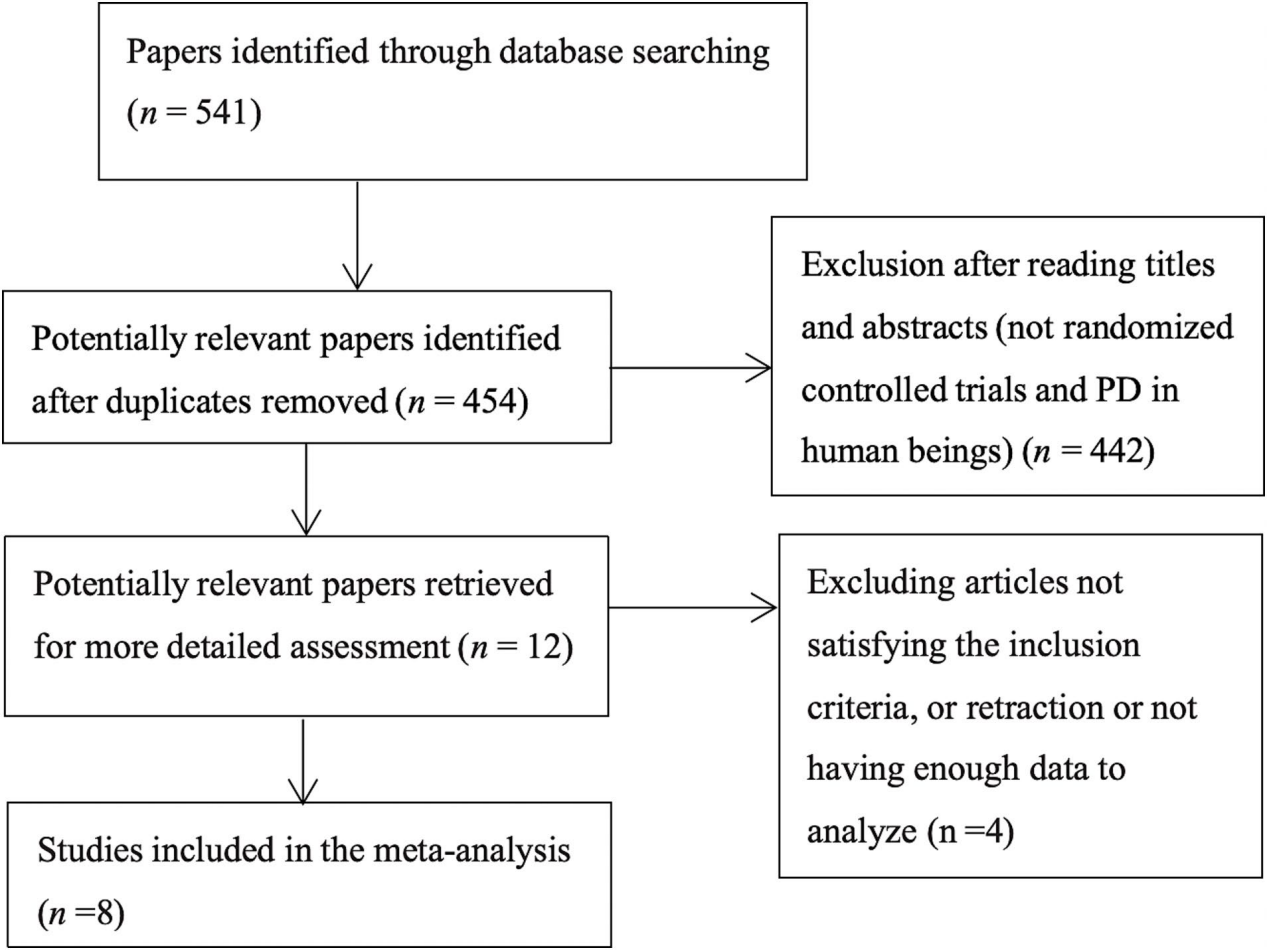
**(A)** Forest plots of the effects of vitamin D supplementation on UPDRS III in patients with Parkinson’s disease. **(B)** Forest plots of the effects of vitamin D supplementation on 10/8 m walk test time in patients with Parkinson’s disease. **(C)** Forest plots of the effects of vitamin D supplementation on timed up and go in patients with Parkinson’s disease. **(D)** Forest plots of the effects of vitamin D supplementation on 6-Minute walking test distance in patients with Parkinson’s disease.

The UPDRS III has been reported in 6 RCTs before and after treatment (4, 23, 30, 33, 34, 36). Vitamin D supplementation had no significant effect on UPDRS III scores in patients with PD (WMD, −0.56; 95% CI, −2.34 to 1.23; *p* = 0.014), and the heterogeneity between studies was significant (*I^2^* = 65.0%) (Figure 3A). The 10/8 m walk test time was reported in two RCTsbefore and after vitamin D treatment (32, 33), which showed no significant effect on the 10/8 m walk test time compared with the application of vitamin D alone (WMD = 0.59, 95% CI = [−0.46, 1.64]; *p* = 0.757), and the heterogeneity of this analysis was non-significant (*I^2^* = 0%) (Figure 3B). TUG was reported in 4 RCTsbefore and after treatment (4, 30, 32, 33), with no significant effect on TUG (WMD: −0.57, 95% CI: −1.45, 0.31, *p* = 0.030), with *I^2^* being 66.4% (Figure 3C). Regarding 6-Minute walking test distance, the trend was significant, and beneficial effects were conferred. Two studies were added to the quantitative analysis for 6-Minute walking test distance (30, 32), which revealed significant walking distance improvements (WMD: 24.85, 95% CI: 6.54 to 43.16, *I^2^* = 40.0%; Figure 3D). Sato et al. (35) study found that Vitamin D supplement inhibited bone mineral density loss and prevented hip and other non-vertebral fractures.

## 4 Discussion

This is the most comprehensive study to assess the relationship between vitamin D supplementation and PD motor symptoms to date. Our pooled meta-data analysis showed that the WMD for 6-Minute walking test distance with vitamin D supplementation was 24.85, suggesting that vitamin D supplementation improve 6-Minute walking test distance by 24.85 meters. Although the motor function evaluation results were not uniform, the UPDRS III, TUG and 10/8 m walk test time results were not statistically significant, vitamin D supplementation appears to be effective for the amelioration of partial motor symptoms in PD.

Although the exact mechanism by which Vitamin D modulates 6-Minute walking test distance is unknown, existing knowledge may provide some clues. Vitamin D supplementation can reduce exercise-induced muscle cell damage (37), thereby preventing falls (22), and attribute up to 22% of the treatment effect to changes in postural balance and up to another 14% to a changes in dynamic balance (4). A meta-analysis indicated that higher levels of serum 25 (OH) D was associated with a lower risk of dementia and AD (38), which may diminish the reaction time in test. In a randomized, double-blind, placebo-controlled study, vitamin D supplementation with vitamin D insufficiency exerted a significant beneficial effect on functional performance, reaction time, and balance after 6 months, and improved neuromuscular or neuroprotective function in older people (39). Meanwhile, a study found vitamin D supplementation improved muscle strength, musculoskeletal function, and balance, and decreased the time needed to perform the TUG test by 11% in community-dwelling elderly hypovitaminosis D patients of both genders (40). Vitamin D supplementation inhibited bone mineral density loss (35), which is higher related to greater muscle strength. Moreover, the effection on muscle strength modulated by specific vitamin D receptors present in human muscle tissue (22, 41). Myocyte-specific vitamin D receptor-null mice showed a distinct muscle phenotype featuring reduced proportional lean mass, reduced voluntary wheel-running distance, reduced average running speed, and reduced grip strength (42), which further supported the beneficial effects of vitamin D on motor function. Further, one study showed that vitamin D and leucine-enriched whey protein oral nutritional supplements resulted in improvements in muscle mass and lower-extremity function among sarcopenic older adults (43). Same as Barichella’s 2019 study (30), vitamin D supplementation also included a whey protein-based nutritional supplement enriched with leucine, moreover, studies suggest the higher 25-hydroxyvitamin D concentrations, the better lower-extremity function in both active and inactive persons aged > or = 60 y (44, 45), which may explain the improvement in 6-min walking test distance after vitamin supplementation in our meta-analysis. However, there was no statistical difference before and after treatment in the UPDRS III, TUG, 10/8 m walk test time, the first reason may be related to the duration and dose of vitamin D administration, as well as oral probiotics or whey proteins at the same time; the second one is the age of PD, which may also be a interfering factor affecting the effectiveness of treatment. In the study of Hiller2018 (4), balance of static and dynamic were measured by Sensory Organization Test using dynamic posturography, the *post hoc* analysis identified evidence of a vitamin D effect upon balance in the younger half of the cohort (mean age 60 years). Therefore, we believes that due to the older age of the subjects in Suzuki et al. (23) and Sato et al. (35), the outcome of UPDRS III has not improved significantly, although the participants in the Habibi et al. (34) study were young, but the duration and dose of vitamin D was significantly lower than in other studies, which may have contributed to the inconsistent results. In the study of Dubose et al. (33), the sample size was small, which may also affect the results. As such, further studies will be necessary to observe vitamin D homeostasis, and conduct long-term, larger-scale randomized controlled trials in the hope of drawing consistent, conclusive conclusions in the future.

We also considered for calcium, probiotics, whey protein, and other substances. In the two studies (4, 30) which treated with vitamin D and calcium, the effects of calcium supplementation on vitamin D were not discussed, limits of ionized calcium was below 1.23 mmol/L. Current research suggests that PD patients should ensure they consume rich calcium and vitamin D to avoid osteoporosis and sarcopenia (46). A study observed an obvious positive association between higher dietary calcium intakes (1249.38 mg/d) and PD risk in men and in ever smokers (47). The decline of dopaminergic neurons due to ROS is linked to elevated calcium levels in the substantia nigra pars compacta (48). So vitamin D deficiency and insufficiency should prompt vitamin D replacement and then maintenance, with or without calcium supplementation according to dietary calcium intake (49). Probiotic supplementation significantly reduced disease severity, alleviated anxiety, and ameliorated gastrointestinal problems among PD patients, but the concurrent use of vitamin D alongside probiotics made it difficult to analyze the role of each of these compounds in the observed results (36). The digestion of whey proteins and leucine leads to rapid increase in essential aminoacid levels, resulting in greater stimulation of muscle protein synthesis (50, 51). Nonetheless, vitamin D drives a positive interaction among the two nutrients in terms of stimulating muscle anabolism and improvement in muscle strength (30, 52).

People over 65 y who have low or no exposure to the sun, gastrointestinal dysfunction, malnutrition, skin atrophy or dark skin that does not produce vitamin D, and renal or liver dysfunction can also affect vitamin D concentrations. People with particular dietary needs (people who avoid nuts, are vegan or have a halal or kosher diet) should be given suitable supplements (21, 53). We suggest a therapeutic dose of 4,000–6,000 for 12 weeks in older PD patients with vitamin D deficiency (≤20 ng/mL) (54) due to poor sun exposure (32) and then given daily supplementation of 2000–5,000 IU/day of vitamin D3 (cholecalciferol) to slow the progression of PD, while also potentially offering additional protection against COVID-19 (55, 56) and play neuroprotective role in PD patients with DBS (57). As for the persist benefits, we suggest ongoing the supplementation for life-long, if feasible (55). The ideal 25 (OH)D3 concentration should be about the physiological level: 40–60 ng/mL (55), at least 30 ng/mL (32).

### 4.1 Limitations

The pooled results of this meta-analysis should be considered in the context of several limitations. First, the number of eligible studies and participants was relatively small. Second, vitamin D supplementation and outdoor activity were assessed using a self-administered diet history questionnaire and self-report questionnaire. The VDR SNPs also varied in each study, which may have affected our results. Third, the potential effects of anti-PD drugs were not explicitly considered in the included studies. Fourth, due to the limited number of eligible studies, we could not perform sex, age, or ethnic subgroup analyses.

### 4.2 Safety

There were no hypercalcinemia and other obvious adverse events associated with vitamin D supplementation in the enrolled studies.

## 5 Conclusion

To our knowledge, this is the most comprehensive study to assess the relationship between vitamin D supplementation and PD motor symptoms to date. The results of our meta-analysis indicated that supplemental vitamin D can prolong 6-Minute walking test distance, improve partial motor symptoms. Vitamin D supplementation may play an active inhibitory role in the mechanisms of PD development. The present results provide evidence for clinicians and other health care professionals to recommend supplemental vitamin D and regular sunlight exposure to prevent disease progression, improve partial motor symptoms during and following treatment.
